# A Functional Role for Modality-Specific Perceptual Systems in Conceptual Representations

**DOI:** 10.1371/journal.pone.0033321

**Published:** 2012-03-13

**Authors:** Louise Connell, Dermot Lynott, Felix Dreyer

**Affiliations:** 1 School of Psychological Sciences, University of Manchester, Manchester, United Kingdom; 2 Decision and Cognitive Sciences Research Centre, Manchester Business School, University of Manchester, Manchester, United Kingdom; 3 Department of Psychology, Leipzig University, Leipzig, Germany; Bielefeld University, Germany

## Abstract

Theories of embodied cognition suggest that conceptual processing relies on the same neural resources that are utilized for perception and action. Evidence for these perceptual simulations comes from neuroimaging and behavioural research, such as demonstrations of somatotopic motor cortex activations following the presentation of action-related words, or facilitation of grasp responses following presentation of object names. However, the interpretation of such effects has been called into question by suggestions that neural activation in modality-specific sensorimotor regions may be epiphenomenal, and merely the result of spreading activations from “disembodied”, abstracted, symbolic representations. Here, we present two studies that focus on the perceptual modalities of touch and proprioception. We show that in a timed object-comparison task, concurrent tactile or proprioceptive stimulation to the hands facilitates conceptual processing relative to control stimulation. This facilitation occurs only for small, manipulable objects, where tactile and proprioceptive information form part of the multimodal perceptual experience of interacting with such objects, but facilitation is not observed for large, nonmanipulable objects where such perceptual information is uninformative. Importantly, these facilitation effects are independent of motor and action planning, and indicate that modality-specific perceptual information plays a functionally constitutive role in our mental representations of objects, which supports embodied assumptions that concepts are grounded in the same neural systems that govern perception and action.

## Introduction

How do we conceive of the world around us? How do we understand linguistic statements about the world? How do we represent objects that are not right in front of our eyes? The question of what constitutes the content of mental representations has long-exercised psychologists and philosophers alike. Extending the views of the British empiricist philosophers (e.g., Hume, Locke, Berkeley), theories of embodied cognition assume that the neural systems we use for conceptual thought (i.e., language processing, problem solving etc.) are grounded in the same neural systems that we use for perception and action [1]–[5]. Reading the words “cinnamon” or “yellow”, for example, leads to increased activations in the same modality-specific neural subsystems engaged when we physically perceive cinnamon or yellowness through our senses of smell and vision [6], [7]. In essence, successfully understanding these words entails partially re-enacting, or perceptually simulating, our prior bodily experiences of cinnamon and yellow.

A growing body of behavioral and neurophysiological evidence supports the notion that conceptual processing engages modality-specific systems (e.g., visual, auditory, motor) and that people automatically simulate perceptual information even when it is superfluous to task requirements [1], [2]. It has been demonstrated that, when people read sentences, they automatically represent perceptual information of mentioned objects such as their shape, color, orientation and motion [8]–[12]. For example, following the sentence “John put the pencil in the drawer” people are faster to recognize the image of a pencil that is subsequently presented in a horizontal orientation onscreen, compared to a pencil that is presented with a vertical orientation. The opposite pattern is found following the sentence “John put the pencil in the cup”, which implies a vertical orientation. From an embodied cognition viewpoint, the reader has perceptually simulated the event in the sentence and so their mental pencil is automatically oriented in a situation-appropriate fashion, thereby facilitating recognition of the object with a matching orientation.

Further to the representation of modality-specific object properties, others have demonstrated action-sentence compatibility effects, where the direction of movement implied by a sentence (e.g., “You handed John the book”), facilitates a congruent body movement (e.g., moving the hand away from the body: [13]). Because the sentence implies movement of the arm, simulating this event leads to effector-specific activations in the motor cortex, which results in faster arm movements in the congruent direction. Such behavioral findings relating to action semantics have been bolstered by findings using neuroimaging techniques. For example, in response to reading action verbs related to different bodily effectors (e.g., lick, pick, kick), somatotopic activations in the motor and pre-motor cortex have been observed [14], [15]. These activations happen so rapidly (<200 ms: [16], [17]) that many have argued that modality-specific perceptual and motor information fundamentally constitutes conceptual content, and therefore plays a functional role in conceptual representations [16]–[18].

Of late, however, a serious challenge has been presented to embodied views of cognition. Several theorists have argued that the patterns of data described above do not support the conclusion that modality-specific perceptual information is constitutive, or functionally required, for the representation of conceptual content [19]–[22]. Instead, it is suggested that any activation in modality-specific brain areas may be nothing more than epiphenomenal; merely reflecting downstream neural activity following initial activation of amodal, abstracted or “disembodied” symbols . For example, any increased neural activation that is observed in the hand area of the pre-motor cortex following the reading of the word “pick” could be the result of spreading activation stemming from prior activation of an amodal symbol of the concept [PICK], with subsequent activations cascading into the motor system. Similarly, representing the appropriate shape or orientation of an object could be achieved by first activating an amodal symbol [PENCIL], which in turn triggers activation in modality-specific areas relating to the relevant object properties. The argument is that behavioral evidence of perceptual simulation or neural activations in sensorimotor and modality-specific brain areas that occur *subsequent* to the presentation of linguistic stimuli may be explicable by initial activations of amodal or disembodied symbols. In this way, the modality-specific and sensorimotor activations may not be functionally required for conceptual content, simply serving as supplementary activations to the core amodal concept.

Here, we propose an alternative approach to answering the question of whether modality-specific perceptual information functionally constitutive of conceptual representations. Much of the evidence in support of embodied representations has emerged from research focussing on motor responses to action-related words and sentences [23]–[28]. For example, people are faster to respond to named objects when their hand posture on an experimental prop matches the grasp aperture afforded by the object (e.g., power grip for “apple”, precision grip for “grape”: [29]). However, as well as being open to the aforementioned criticisms of downstream activation, this approach conflates motor information with perceptual information, and, crucially, leaves open the possibility that observed effects may be due in part to planning and executing a motor response with the relevant effector. In the present study, we focussed instead on the modalities of touch and proprioception, and tested whether perceptual information from these modalities does indeed play a functional, constitutive role in conceptual representations. We used a behavioral paradigm where participants received concurrent perceptual stimulation while completing a conceptual task in order to determine whether such perceptual stimulation impacts on conceptual processing in a manner consistent with embodied views of cognition, but inconsistent with “disembodied” views of cognition. As a conceptual task, participants made semantic judgements of object size comparison, where they decided which was the bigger or smaller object of a pair of named objects. Our approach separated tactile and proprioceptive representations from action planning by measuring the speed of participant voice responses (where the mouth is a non-relevant effector for the objects being judged), which obviated the need for responses that required overt actions using object-relevant effectors.

While people can retrieve visual information about the size of objects [30]–[34]), another source of information about object size comes from physical interaction; the arms, hands and fingers feed back tactile and proprioceptive information when contact is made with an object. Embodied cognition views argue that this kind of body-specific information plays an important functional role in conceptually representing objects because cognition is grounded in the same neural systems that govern perception and action. For example, in order to decide whether a *wallet* or a *key* is bigger, a strong interpretation of such theories would assume that past experiences across various modalities – visual, motor, tactile, proprioceptive, etc. – will be partially re-enacted, and the resulting simulations of wallet and key will then be compared. Simulating non-visual information, however, depends on being able to interact physically with the object in question. While a *wallet* or *key* can be picked up and spanned by the hands, a *mansion* or a *cottage* cannot offer the same opportunities for tactile and proprioceptive interactions. Thus, from an embodied perspective, providing concurrent tactile and proprioceptive stimulation should influence conceptual processing of manipulable objects only, but in what way?

Previous work has shown that bodily feedback can facilitate cognitive processing by directing attentional resources to relevant neural systems. For example, when the mouth is unconsciously pulled into a smiling expression by holding a pen between the teeth, people find cartoons funnier [35] and are quicker to understand sentences that describe pleasant or happy situations (e.g., *You and your lover embrace after a long separation*: [36]; see also [37]–[39] for examples of how facial immobilisation can interfere with processing such stimuli). Similarly, slumping in a chair makes it easier for people to retrieve sad or negative memories [40], [41], while lying down speeds up people's recall of visiting the dentist [42]. Even at a modality-specific level, perceptual primes have been shown to facilitate simulation in that modality [43], providing attentional demands in that modality are not too high [44]. Based on such work, we expected bodily feedback from touch and arm/hand positioning to direct attention to the modality in question (i.e., touch and proprioception) and hence facilitate the speed of simulating conceptual information in those modalities. Accordingly, we applied tactile or proprioceptive stimulation either to the hands as critical object-relevant feedback, or to the feet as control object-irrelevant feedback, while people judged pairs of objects that were either small and manipulable or large and nonmanipulable. Since the simulations formed during conceptual processing should be based on experience in all relevant modalities, we predicted that people would be faster to make conceptual size comparisons during tactile and proprioceptive stimulation, but that such facilitation would be limited to (a) stimulation of the hands, and (b) objects of a physically manipulable size. By contrast, a disembodied view would predict that providing sensorimotor inputs concurrently with conceptual processing will either have no impact on conceptual processing or will impact only generally on processing (i.e., with a general facilitatory or inhibitory effect), but with no differential effects on the processing of manipulable and nonmanipulable objects.

**Figure 1:**
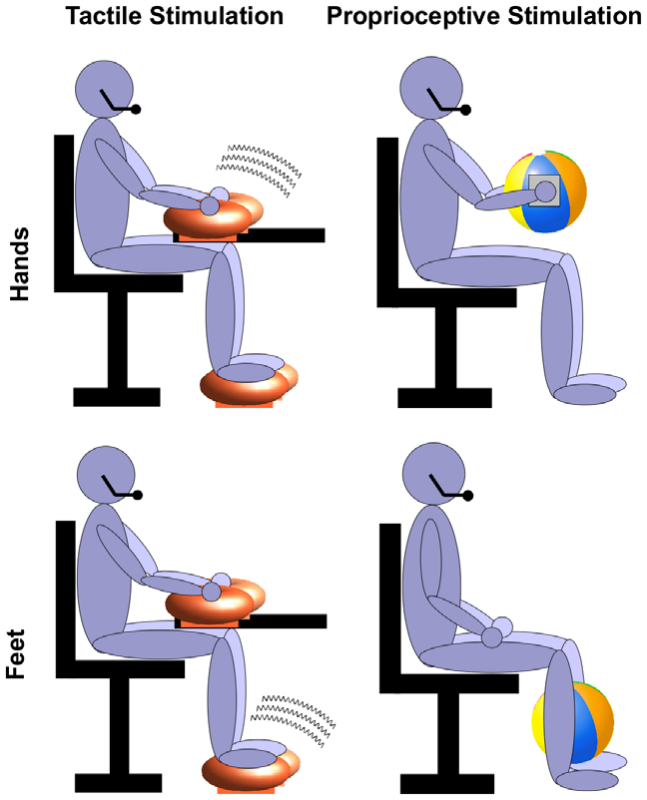
Schematic of perceptual stimulation to the hands (critical condition) or feet (control condition), showing participant receiving tactile stimulation from vibrating cushions, or proprioceptive stimulation from holding a 30 cm diameter beachball.

In the first experiment, people performed the size comparison task while receiving tactile stimulation from vibrating cushions. By resting hands on cushions, participants also received constant proprioceptive stimulation to the hands and arms in both critical and control conditions. However, in the critical condition, where the hand cushions were vibrating, participants experienced vibrotactile feedback [45] to the skin on palm and fingers which was absent during the control condition (see Figure 1 for a schematic of the conditions). In the second experiment, we manipulated proprioceptive information by having participants passively hold an object (i.e., an inflated beachball) while they performed the object size comparison task. Here, participants received constant tactile stimulation to the hands in both critical and control conditions (i.e., skin on palm and fingers was in continuous lightly-pressured contact with a flat surface). Crucially, holding a lightweight beachball in the critical condition meant that participants received isometric proprioceptive feedback from the hands and arms (i.e., stable muscular tension during passive holding, without change in the length of muscle fibers), which was absent during the control condition. In both experiments, hand- and foot-stimulation took place in two counterbalanced blocks. The pairs of objects being compared were either both manipulable (i.e., of small size and can be held in one hand, such as *wallet*, *key*, *coin*) or both nonmanipulable (i.e. of large size and greater than arms' width, such as *mansion*, *car*, *cottage*).

## Results

**Figure 2:**
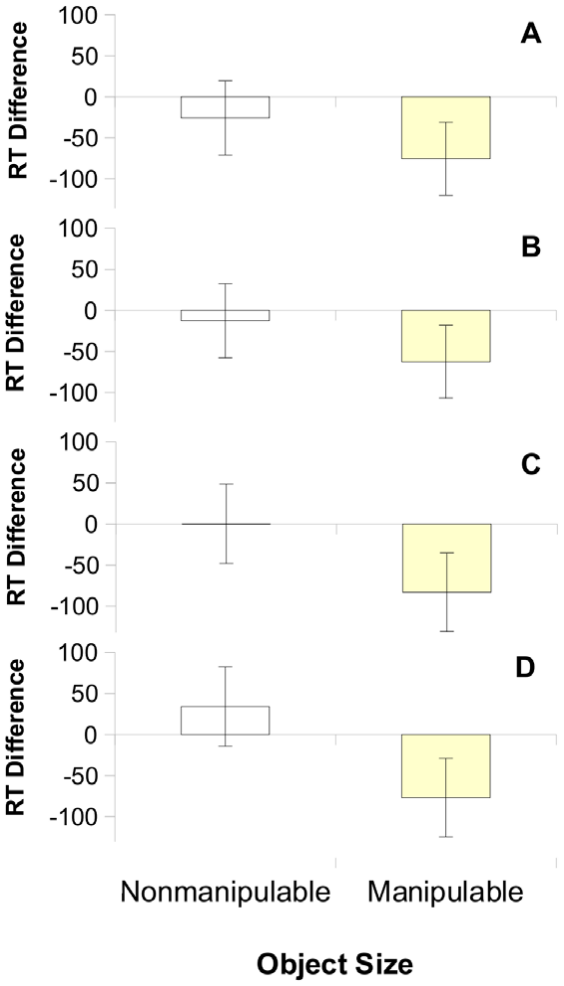
Size judgement effects (in ms) for tactile stimulation (a: bigger judgements; N = 20, b: smaller judgements; N = 21) and proprioceptive stimulation (c: bigger judgements; N = 23, d: smaller judgements; N = 22), showing consistent facilitation for small, manipulable objects but not for large, nonmanipulable objects. RT difference was calculated by subtracting judgement times in the control foot-stimulation condition from judgement times in the critical hand-stimulation condition. Error bars show 95% confidence intervals of the difference between means.

Results are presented graphically in Figure 2A–D, with response times and accuracy per condition reported in Table 1. Overall, findings from linear mixed model analyses were as predicted. Relative to foot-stimulation controls, perceptually stimulating the hands facilitated participants' judgements for manipulable objects alone [tactile hand *M* = 1459 ms, *SE* = 47 ms; tactile foot *M* = 1528 ms, *SE* = 47 ms: *F*(1, 3520.1) = 18.39, *p*<.0001, *r* = .072; proprioceptive hand *M* = 1533 ms, *SE* = 48 ms; proprioceptive foot *M* = 1612 ms, *SE* = 48 ms: *F*(1, 3795.2) = 22.06, *p*<.0001, *r* = .076]. By contrast, neither tactile nor proprioceptive stimulation influenced the processing of nonmanipulable objects (tactile hand *M* = 1576 ms, *SE* = 47 ms; tactile foot *M* = 1595 ms, *SE* = 47 ms: *p* = .243; proprioceptive hand *M* = 1641 ms, *SE* = 48 ms; proprioceptive foot *M* = 1624 ms, *SE* = 47 ms: *p* = .326).

**Table 1:** Mean response times (ms) and accuracy levels (%), with standard errors in parentheses, for all factor combinations in both tactile and proprioceptive stimulation experiments.

			Tactile stimulation		Proprioceptive stimulation	
Judgement type	Object size	Stimulation position	RT	Accuracy	RT	Accuracy
Bigger	Manipulable	Hands	1450 (64)	97.8 (1.1)	1504 (63)	95.6 (1.2)
		Feet	1525 (64)	97.1 (1.3)	1587 (63)	95.4 (1.6)
	Nonmanipulable	Hands	1517 (64)	90.9 (1.6)	1540 (63)	88.9 (1.6)
		Feet	1543 (64)	89.9 (1.9)	1540 (63)	90.5 (1.6)
Smaller	Manipulable	Hands	1468 (63)	94.9 (1.1)	1561 (64)	94.8 (1.2)
		Feet	1531 (62)	96.1 (1.2)	1638 (64)	92.8 (1.6)
	Nonmanipulable	Hands	1635 (63)	92.5 (1.6)	1742 (64)	92.7 (1.7)
		Feet	1647 (63)	91.5 (1.9)	1708 (64)	91.4 (1.7)

Effects were robust regardless of the direction of the object comparison. For tactile stimulation, critical facilitation for manipulable objects emerged both when participants judged which object was bigger (Figure 2A): F(1, 3509.2) = 10.88, *p* = .001, *r* = .056; and which object was smaller (Figure 2B): *F*(1, 3528.7) = 7.65, *p* = .006., *r* = .047. Neither bigger nor smaller judgements showed any difference for nonmanipulable objects, *p* = .276 and *p* = .580, respectively. An identical pattern emerged for proprioceptive stimulation, with facilitated processing of manipulable objects for both “which is bigger” (Figure 2C): *F*(1, 3791.9) = 12.27, *p*<.001, *r* = .057; and “which is smaller” comparisons (Figure 2D): *F*(1, 3794.0) = 9.94, *p* = .002, *r* = .051; but no facilitation for nonmanipulable objects, “bigger” judgements (*p* = .990); “smaller” judgements, (*p* = .172).

Overall, people responded more quickly during stimulation to the hands than stimulation to the feet [tactile *F*(1, 3519.9) = 14.67, *p*<.001, *r* = .064; proprioceptive *F*(1, 3795.6) = 6.71, *p* = .010, *r* = .042], and manipulable objects were judged faster than nonmanipulable [tactile *F*(1, 96.1) = 7.54, *p* = .007, *r* = .270; proprioceptive *F*(1, 97.7) = 3.03, *p* = .085, *r* = .173], with the critical size by position interaction emerging under both stimulation modalities [tactile *F*(1, 3519.7) = 4.66, *p* = .031, *r* = .036; proprioceptive *F*(1, 3795.6) = 15.95, *p*<.0001, *r* = .065]. Judgement type had no main effect (tactile *p* = .455; proprioceptive *p* = .146), but did interact with object size [tactile *F*(1, 3510.4) = 18.67, *p*<.0001, *r* = .073; proprioceptive *F*(1, 3790.6) = 29.25, *p*<.0001, *r* = .088] because “which is smaller” judgements were generally faster for manipulable objects than nonmanipulable, while “which is bigger” judgements made little difference. There were no other interactions, all *p*s>.4.

Combined analysis of both experiments revealed similar effect sizes for both tactile and proprioceptive stimulation: the critical interaction (size by position) does not in itself interact with stimulation modality (i.e., touch, proprioception), F(1, 7403.1) = 2.23, p = .136, nor does it produce a four-way interaction with stimulation modality and judgement type (i.e., bigger, smaller comparisons), p>.2.

## Discussion

We found that stimulating the hands with tactile vibrations or proprioceptive isometric positioning made it easier for participants to compare small, manipulable objects like *coins* or *frisbees.* Objects that were too large to be physically manipulable, like *cars* and *windmills*, were unaffected by either tactile or proprioceptive stimulation. These findings support the idea that size representations of manipulable objects include modality-specific information about touch and position that specifically relate to the hands, whereas size representations of nonmanipulable objects lack such information. This modality-specific, body-specific facilitation effect was independent of the direction of the size comparison. While there has been some agreement that object size representations have a strong visual component, distinct from amodal propositional representations such as [size:5 cm] or [size:huge], with evidence coming from numerous behavioral and neuroimaging studies [30]–[34], [46]–[48], the current findings of distinct tactile and proprioceptive effects on size comparisons provides the first evidence that the senses of touch and proprioception uniquely and separably contribute to object representations. Furthermore, by requiring vocal responses, they confirm that tactile and proprioceptive object representations are independent of planning an associated action with a relevant effector. In short, this study shows that modality-specific tactile and proprioceptive perceptual information has a functionally constitutive role to play in the conceptual representation of objects that is consistent with body-specific experience of such objects. The pattern of results is difficult to square with an account that requires the initial activation of an amodal symbolic representation, with only downstream, incidental activation of perceptual information. We return to this point below.

The issue remains as to what mechanism allows for such facilitation effects. The answer to this question lies in the nature of the representations we construct for objects in the world. From an embodied or grounded cognition perspective, it is through our situation-specific interactions with objects themselves that we build up their representations. Crucially, these representations include the modality-specific information that is perceived during experience (e.g., [49], [50]). So, holding a cup will stimulate, for example, visual, tactile and proprioceptive senses, leading to activation of a particular set of cell assemblies, or network of distributed neural representations, all of which constitute the cup's percept. Through Hebbian learning, these cell assemblies become associated with a verbal code (i.e., the word “cup”) which co-occurs over time with these perceptual activations [51], [52]. This set of multimodal activations is then re-activated, or simulated [1], [2], [53]–[55] during conceptual tasks such as size comparisons, property-verifications or sentence comprehension. In the present studies, the provision of tactile or proprioceptive stimulation to the hands means that cell assemblies associated with small, manipulable objects like cups are already partly activated due to a partial overlap between their activations and the concurrent sensory input. Thus, some of the modality-specific information that is needed to perform the perceptual simulation of the objects in the size-comparison task has already been activated. In other words, holding a beachball leads to a patterns of neural activation that overlaps with the proprioceptive simulation of a small, manipulable object, and it is this overlapping activation that leads to facilitation in judging that object's size. As larger objects do not afford such physical interactions, they provide little or no proprioceptive information during perception. During conceptual tasks, therefore, providing proprioceptive stimulation cannot provide a facilitatory effect for the processing of large, nonmanipulable objects.

Returning to the key issue of whether these results can be explained by a downstream activation mechanism cascading from an amodal or abstracted symbolic representation, one could assume that there is an amodal symbol for “cup” and we find that, due to tactile stimulation to the hands, a person's conceptual processing related to “cup” is facilitated. One could argue that the tactile stimulation merely increases speed of access to an abstract symbol in the conceptual task, which is subsequently manifested in faster response times. However, this argument can only work if the same tactile sensory stimulation leads to equivalent facilitation of amodal symbols for all object types (i.e., both manipulable and nonmanipulable objects), but this is not what happens. Small, manipulable objects are differentially affected relative to large, nonmanipulable items. An amodal symbol for “cup” cannot encode these differences without the inclusion of perceptual information related to the object's size properties. To achieve such a distinction, a committed amodalist could propose a hybrid of downstream and upstream activation, where stimulation to the hands preferentially spreads activation upstream to a symbol for “manipulable”, which in turn spreads activation to the symbols for all manipulable objects, thus meeting the downstream activation of the symbol for “cup” when the word is presented onscreen. However, this argument founders with closer inspection of the results. People were faster to make “which is smaller” judgements about manipulable objects than nonmanipulable objects, while this difference did not occur for “which is bigger” judgements (i.e., judgement type interacted with object size in both tactile and proprioceptive studies). In amodal terms, this effect equates to a close relationship between a “small” symbol (activated by the relevant judgement task) and a “manipulable” symbol (activated by hand stimulation), whereas an equivalent “big” symbol (activated by the relevant judgement task) has no such relationship with “manipulable”. If hand stimulation preferentially activated a “manipulable” symbol in a way that foot stimulation did not, one would therefore expect such hand stimulation to mediate the relationship between “small” and “manipulable” symbols (but to have little effect on the nonexistant relationship between “big” and “manipulable”). No such effect occurred (i.e., there is no three-way interaction between judgement type, object size and stimulation position in either study). Finally, one could argue that asking participants “which is smaller” activated an amodal symbol for “small”, which in turn spread activation to a range of related symbols for manipulable objects and thus facilitated their processing. Again, this argument founders with examination of the results: the critical facilitation effect means that manipulable objects were only processed more quickly when the participant's hands (as opposed to their feet) were perceptually stimulated. Furthermore, the same facilitation effect occurred regardless of comparison direction: it did not matter whether participants were judging which object was bigger or smaller. Thus, potential symbolic associations between the stimulation position/judgement task and items cannot account for the pattern of results. An alternative view is that the object representation comprises perceptual information relating to the physical, perceptual properties of the object. In short, modality-specific perceptual activations play a functional role in conceptual representation, and do not simply serve as epiphenomenal supplements to a core amodal concept.

We have previously shown that modality-specific perceptual information is automatically represented in conceptual processing of words relating to touch, vision, taste, smell and hearing [49], [56]. Other studies have shown various modality-specific effects for the same set of five basic senses (e.g., [8], [9], [43], [50], [57], [58]). The present work is the first demonstration that proprioception can be added to the list of automatically-represented perceptual modalities in conceptual tasks, such as the size-comparison task employed here. The knowledge that perceptual information informs our conceptual representations across a range of modalities, and that sensorial feedback impacts on these representations, will play a crucial role in the further development of embodied theories of cognition. Moreover, because of the tight coupling between the perceptual and conceptual systems, it is possible that linguistic and other conceptual tasks may be beneficial in developing novel, non-invasive therapeutic approaches (e.g., [59], [60]) for treating perceptual and sensorimotor deficits, such as in stroke rehabilitation [61].

In conclusion, the present findings enhance our understanding of embodied conceptual representations, demonstrating that people can “hold” something in the mind's hands by simulating modality-specific information captured during perceptual experience. Furthermore, while vision is a useful and fundamental means of perceiving and representing objects, the importance of bodily feedback provided by touch and proprioception should not be underestimated as they offer valuable means of conceptualizing the world around us. The current pattern of results is not consistent with a disembodied view of conceptual representations that confines perceptual information to an epiphenomenal role. Rather, it seems that modality-specific, perceptually grounded representations that are functionally constitutive of conceptual content are needed to explain the relationship between perceptual stimulation, bodily feedback and conceptual activation.

## Materials and Methods

### Participants

The studies were approved by the Research Ethics Committee of the School of Psychological Sciences, University of Manchester and conform to the principles outlined in the Declaration of Helsinki. For each study, participants gave written informed consent prior to participation. Eighty-six volunteers from the University of Manchester took part for course credit or a £3 reward. Forty-one participants (23 female, 18 male; Mean age = 23.1 years) completed the tactile stimulation task (bigger judgements *N* = 20, smaller judgements *N* = 21; see procedure for details), while forty-five (26 female, 19 male; Mean age = 22.9 years) completed the proprioception stimulation task (bigger judgements *N* = 23, smaller judgements *N* = 22). All participants were naïve to the experiment, had normal or corrected-to-normal vision, were fluent in English, and had no mobility or reading impairments.

### Materials

Stimuli for the size comparison task consisted of 100 pairs of object names: 50 pairs of small-size, manipulable objects (e.g., COIN:FRISBEE, ALMOND:PEAR, CRAYON:PEN) and 50 pairs of large-size, nonmanipulable objects (e.g., CAR:VAN, CAMEL:COW, MANSION:COTTAGE). Both items in each pair were from the same category (both buildings, both fruits, both artifacts, etc.) with one object in each pair bigger than the other. In a pretest, three independent raters correctly classified the larger/smaller item of each pair in 100% of cases. There were no differences in word length between bigger and smaller items in each pair, nor between big and small items in general (*p*s>.3). Four counterbalanced lists of stimuli were created (each with 25 small and 25 large pairs), to ensure that all items would appear in both the hand- and foot-stimulation blocks, as well as appearing on both the left- and right-hand positions onscreen. This counterbalancing ensures that all items appear in all conditions.

In order to ensure our manipulation was not confounded by lexical associations, we calculated the conditional probability of encountering each object name given the name of the body part stimulated for that block. For example, using the Web 1T 5-gram corpus [62], which consists of over a trillion words culled from Google indices, the frequency of “hand” and “coin” was obtained when zero to three words occurred between them, and then divided by the frequency of the word “hand”. Analysis of variance showed that the word “hand” was marginally more related to all object names than the word “foot”, F(1, 198) = 2.84, p = .093, *r* = .119, but there was no interaction with object size (p = .768), and no effect of object size itself (p = .448). In other words, “hand” was just as likely to predict the names of manipulable objects (M = 0.013%) as nonmanipulable objects (M = 0.018%), as was “foot” (M = 0.007% and M = 0.010%, respectively), and so lexical priming could not give rise to our predicted pattern of effects.

### Procedures

In the tactile stimulation paradigm, participants removed their shoes and sat in a chair in front of a computer screen. The experimenter then placed a massage cushion under each hand and foot (see Figure 1) and participants remained in this position for the duration of the experiment. The hand cushions vibrated to provide tactile stimulation in the critical block, while the foot cushions vibrated to provide equivalent sensory distraction in the control block. While vibration can sometimes affect proprioception, the vibrotactile apparatus in the present experiment was unlikely to do so both because the vibration frequency (67 Hz) was below that at which Pacinian corpuscles (cutaneous sensory receptors that have been found to contribute to proprioception: see e.g., [63]) consistently respond, and because vibration in the present study was not applied directly over tendons in the elbow or ankle in order to vibrate muscle spindles (and thus create proprioceptive illusions of movement: e.g., [64], [65]) but rather was applied to the glabrous skin of the hand and foot. In the proprioception stimulation paradigm, participants also sat in front of a computer screen, but in the hand stimulation condition participants held a beachball of 30 cm diameter in front of their bodies at chest height, without letting the ball touch their knees or the table in front, which positioned their hands a constant distance apart (see Figure 1). Squares of stiff card were attached to both sides of the beachball, and participants placed their hands flat on the card, secured by rubber finger loops, to prevent the curvature of the ball providing unwanted shape information. The feet were kept flat on the ground for the duration of the block. In the foot stimulation (control) block, participants held the beachball between their lower legs, as far down as possible without letting the ball touch the ground. The hands were placed flat on the thighs, with arms relaxed, for the duration of this block. Holding the beachball, rather than simply holding hands/feet apart in isolation, ensured that participants kept their hands/feet at a stable distance apart for the duration of the experiment and meant that participants received isometric proprioceptive feedback (i.e., stable muscular tension during passive holding, without change in the length of muscle fibers) from the hands and arms, which is absent during the control condition.

For the size comparison task, each pair of object names was presented in capital letters, 4 cm apart in the center of the screen (left-right order counterbalanced), separated by a colon. Once a vocal response had registered, the screen blanked for 1500 ms before the next trial. Participants received automatic feedback if their responses were outside the valid range (250–3000 ms). Trials were randomly presented within each block, with different randomizations for each participant. Since size comparison is bidirectional, participants were randomly allocated to make either bigger or smaller judgements. In other words, Participant A would always judge which object of a pair was bigger, while Participant B would always judge which object was smaller. Participants were told they would see the names of two objects onscreen and that they should state aloud, as quickly as possible, which item was bigger (or smaller) in size. If participants were unfamiliar with any presented words, they were asked to say so and the trial was marked as invalid. To record responses, participants wore a head-mounted unidirectional microphone. Response times were measured from the appearance of the object names to the onset of the vocal response. Any trials where disfluencies (e.g., coughs, hesitancies) triggered the microphone were marked as invalid. A practice session of ten trials preceded the main experiment to familiarize participants with the task and allow for microphone calibration.

### Design & Analysis

Response time data in valid trials were analyzed separately for tactile and proprioceptive studies using linear mixed models, which allows simultaneous analysis with participants and items as crossed random factors [66], [67]. Crossed fixed factors were object size (manipulable, nonmanipulable) and stimulation position (hands, feet) as within-participant manipulations, and judgement type (bigger, smaller) as between participants. All condition means presented in the text are estimated marginal means in milliseconds, and effect size *r* is calculated from *F* (where numerator df = 1) as per *t* [68]. Response times that were greater than 2.5 standard deviations from a participant's mean per condition were classed as outliers and removed prior to analysis. This resulted in a loss of 2.47% from the tactile study and 2.72% from the proprioception study.

Accuracy levels were high and no participants were excluded due to low accuracy scores. There was no evidence of speed-accuracy tradeoff. Analysis of mean accuracy per participant showed that manipulable objects were processed *more* accurately than nonmanipulable ones (tactile manipulable *M* = 96%, nonmanipulable *M* = 91%, *F*(1, 39) = 40.23, *p*<.0001, *r* = .713; proprioceptive manipulable *M* = 95%, nonmanipulable *M* = 91%, *F*(1, 43) = 28.49, *p*<.0001, *r* = .631), and this effect did not vary by hand/foot stimulation (tactile *p* = .418; proprioceptive *p* = .368).

### Debriefing

On completion of the task, participants were asked whether they thought they understood the purpose of the study. In no case did a participant mention anything about object representations or perceptual information, nor did any participant suspect the true reason for the tactile or proprioceptive manipulations.
